# Association between food and nutrition insecurity with cardiometabolic risk factors in childhood and adolescence: a systematic review

**DOI:** 10.1016/j.rppede.2016.01.006

**Published:** 2016

**Authors:** Naruna Pereira Rocha, Luana Cupertino Milagres, Juliana Farias de Novaes, Sylvia do Carmo Castro Franceschini

**Affiliations:** Departamento de Nutrição e Saúde, Universidade Federal de Viçosa (UFV), Viçosa, MG, Brazil

**Keywords:** Food and nutrition security, Diabetes mellitus, Hypertension, Metabolic syndrome, Stress and dyslipidemia

## Abstract

**Objective:**

To address the association between food and nutrition insecurity and cardiometabolic risk factors in childhood and adolescence.

**Data source:**

Articles were selected from the Medline, Lilacs and SciELO databases with no publication date limit, involving children and adolescents, using the descriptors: food and nutrition security, diabetes mellitus, hypertension, metabolic syndrome, stress and dyslipidemia. The terms were used in Portuguese, English and Spanish. The search was carried out systematically and independently by two reviewers.

**Data synthesis:**

Exposure to food insecurity during childhood and adolescence ranged from 3.3% to 82% in the selected publications. Exposure to food insecurity was associated with stress, anxiety, greater chance of hospitalization, nutritional deficiencies, excess weight and inadequate diets with reduced intake of fruits and vegetables and increased consumption of refined carbohydrates and fats.

**Conclusions:**

Food and nutrition insecurity was associated with the presence of cardiometabolic risk factors in the assessed publications. Childhood and adolescence constitute a period of life that is vulnerable to food insecurity consequences, making it extremely important to ensure the regular and permanent access to food. Because this is a complex association, some difficulties are found, such as the synergy between risk factors, the assessment of heterogeneous groups and extrapolation of data to other populations, in addition to the influence of environmental factors.

## Resumo

**Objetivo:**

Abordar a associação entre insegurança alimentar e nutricional e fatores de risco cardiometabólicos na infância e adolescência.

**Fontes de dados:**

Os artigos foram selecionados pelas bases de dados Medline, Lilacs e SciELO, sem limite de data de publicação. Envolveram crianças e adolescentes e foram usados os descritores: segurança alimentar e nutricional, diabetes melito, hipertensão, síndrome metabólica, estresse e dislipidemia. Os termos foram usados em português, inglês e espanhol. A busca foi feita de forma sistemática e independente por dois revisores.

**Síntese dos dados:**

A exposição à insegurança alimentar no período da infância e adolescência variou de 3,3% a 82% nas publicações selecionadas. A exposição à insegurança alimentar esteve associada a estresse, ansiedade, maiores chances de internações hospitalares, deficiências nutricionais, excesso de peso e consumo de dietas inadequadas com ingestão reduzida de frutas e hortaliças e aumento do consumo de carboidratos refinados e gorduras.

**Conclusões:**

A insegurança alimentar e nutricional esteve associada à presença de fatores de risco cardiometabólicos nas publicações avaliadas. A infância e adolescência constituem uma fase da vida vulnerável às consequências da insegurança alimentar, o que torna de extrema importância a garantia do acesso regular e permanente aos alimentos. Por ser essa associação complexa, algumas dificuldades são encontradas, tais como a sinergia existente entre os próprios fatores de risco, a avaliação de grupos heterogêneos e a extrapolação dos dados para outras populações, além da influência dos fatores ambientais.

## Introduction

The approach to food and nutrition insecurity (FNiS) has gained prominence in developed and developing countries. The concept of food and nutrition security (FNS) was established by the Second National Conference on Food Security held in 2004 in Brazil and consists in the right of all individuals to regular and permanent access to quality food in sufficient quantity, without compromising the access to other essential needs, based on food practices that promote health, respect cultural diversity and that are environmentally, economically and socially sustainable.^1^ Situations that include the violation of any of these items constitute FNiS.

One can observe the association of FNiS not only in the context of low birth weight and/or presence of deficiency diseases, as widely debated by researchers, but also related to a "new" association of the topic with the presence of cardiometabolic risk factors developed as early as the childhood and adolescence periods, such as obesity, insulin resistance, type II diabetes, systemic arterial hypertension, dyslipidemia and inflammation.^2^^-^ 4

These risk factors can be classified into traditional (modifiable or not) and non-traditional. Traditional non-modifiable factors include age, gender and family history of premature cardiovascular disease, while the modifiable ones include dyslipidemia, arterial hypertension, type II diabetes, smoking, physical inactivity and excess weight. The so-called non-traditional factors encompass the assessment of some cardiometabolic risk markers, such as inflammatory cytokines, C-reactive protein, interleukin-6, leptin and adiponectin.^5^

Some studies have shown positive associations between the presence of FNiS and poorer health status in children and adolescents.^6^^-^ 8 Among these outcomes, we emphasize behavioral, psychosocial and developmental problems, with greater prevalence of acute and chronic diseases.^9^ However, the precise mechanism by which FNiS negatively affects the health status of this group is yet to be elucidated.^10^

Based on this perspective, after verifying the scarcity of Brazilian studies on this topic, a systematic review was performed to assess whether food and nutrition insecurity is associated with the presence of cardiometabolic risk factors in childhood and adolescence, with the aim of providing subsidies for public health interventions. The main elements to be modified regarding this issue will help plan future intervention studies for children and adolescents in a FNiS situation.

## Method

The search strategy included the search for articles in electronic databases. The Medline (National Library of Medicine, USA) via PubMed, Lilacs (Latin American and Caribbean Health Sciences) and SciELO (Scientific Electronic Library Online) electronic databases were used in the search. Article identification and selection in all searched databases were performed simultaneously by two researchers for a month, between August and September 2014.

The descriptors used were: food and nutrition security, diabetes mellitus, hypertension, metabolic syndrome, stress and dyslipidemia. All descriptors were used in Portuguese, according to the Health Sciences Descriptors (DeCS), and English, according to Medical Subject Headings (MeSH). The terms were also used in Spanish to encompass a greater number of studies published in the area. The food and nutrition security descriptor was combined with other descriptors through the use of Boolean operators represented by the connector terms AND, OR and NOT. Therefore, the following combinations were used: food and nutrition security AND diabetes mellitus, food and nutrition security OR diabetes mellitus and food and nutrition security NOT diabetes mellitus. These combinations were always used associating the descriptor food and nutrition security to the others.

The risk factors for cardiometabolic diseases (diabetes mellitus, hypertension, metabolic syndrome, stress and dyslipidemia) were used as descriptors, as chronic diseases are generally not present in children and adolescents. However, the presence of risk factors that lead to the development of these diseases can be identified.

The review searched for studies that assessed children and adolescents, because this one of the periods of greatest vulnerability to food deprivation and occurrence of disorders related to growth and physiological development and that can occur together with health problems.^11^^,^ 12 Therefore, the review included articles that associated FNiS to at least one of the cardiometabolic risk factors in children and/or adolescents.

The exclusion criteria included studies on adults, the elderly, pregnant women, groups of children/adolescents with low birth weight, those with congenital diseases, as well as the literature review and/or systematic review articles, dissertations, theses, consensus and documents from national and international organizations, repeated articles in different databases and published in other languages rather than Portuguese, English and Spanish.

The identification and selection of articles in the databases were performed by two researchers, independently and systematically, who carried out the initial selection by analyzing the titles of publications found through the use of descriptors and, subsequently, through the abstracts obtained by electronic search. After the selection of publications through the titles and abstracts, a new analysis was carried out by the two researchers, who consensually determined which studies should be read in full and included in the review. The references of the selected articles were screened, aiming to include other studies of potential interest.

The assessment of food and nutrition insecurity in the selected articles was considered based on the data obtained through questionnaires and/or structured questions, applied to the children, adolescents or parents/guardians and/or based on the social and economic data of the assessed families. FNiS was identified by reading the publications in search of situations that addressed the presence of the physical sensation of hunger and/or food insecurity due to reasons related to income and/or interruption in the feeding patterns, resulting from the lack of food and/or food intake assessment.

For the methodological assessment of publications, we aimed to answer the question "Is food and nutrition insecurity associated with the presence of cardiometabolic risk factors in children and adolescents?" Considering the question, all associations between FNiS and nutritional status, biochemical parameters, overall health status and food intake were explored, as addressed by the identified studies.

## Results

**Figure 1:**
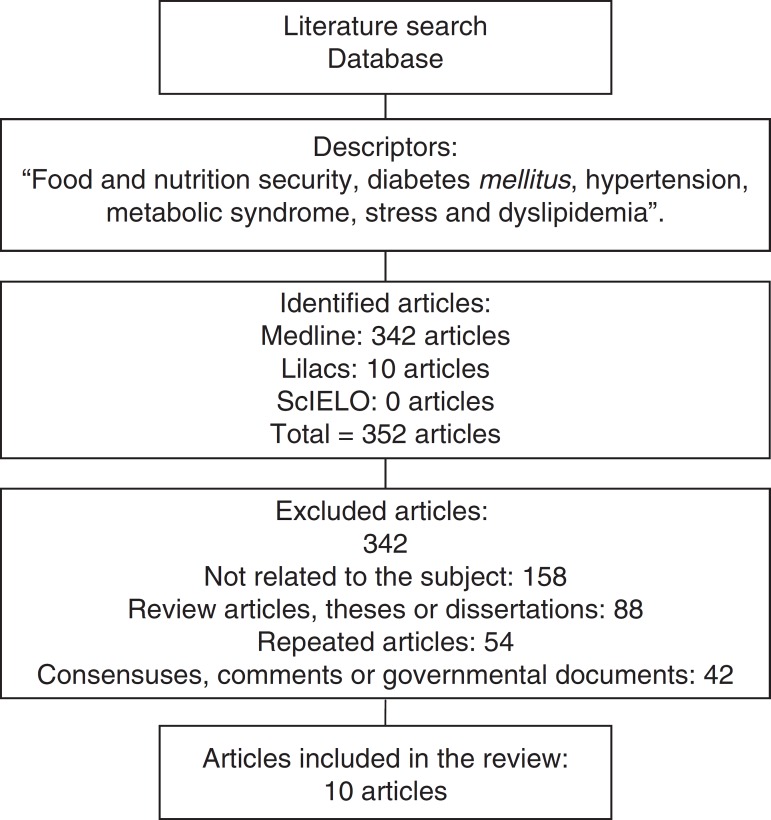
Articles selected for content assessment.

The search for the descriptors resulted in the identification of 352 articles in the area of interest. A total of 342 publications were excluded (158 publications were not related to the topic, did not meet the objective of the study, did not evaluate children or adolescents, 88 were review articles, theses or dissertations, 54 articles were repeated in different databases, 42 were related to consensuses, expert commentaries or government agencies documents). Only 10 articles met the inclusion criteria, of which three were obtained through the search performed in the references of the preselected publications (Fig. 1).

Of the assessed studies, eight articles had a cross-sectional design and all had international samples from North America, Europe and Asia. No national article that assessed the intended subject was identified through the search for the descriptors.

The approach of the association of FNiS with some cardiometabolic risk factor is relatively new in science. The researchers’ interest in FNiS related to chronic health status alterations during childhood and adolescence can be observed after 2002^13^ (Table 1). None of the studies addressed the association between the presence of FNiS and non-traditional cardiometabolic risk factors (inflammatory cytokines, C-reactive protein, interleukin-6, leptin and adiponectin).

**Table 1:** Evaluation of publications on food and nutrition insecurity and cardiometabolic risk factors in childhood and adolescence.

Author/year	Design	Sample	Age	Nutrition variables	Nutritional Status Classification	Tool to assess food and nutrition security
Weinreb et al., 2002^13^	Cross-sectional	408 preschoolers, children and adolescents	2.5-17 years	Do not assess	Do not perform	Community Childhood Hunger Identification Project and direct questions for children older than 9 years
Cook et al., 2004^7^	Cross-sectional with cohort.	11,539 children's caregivers	Children <36 months	Weight and height	Do not report	U.S. Household Food Security Scale (U.S. HFSS)
Molcho et al., 2006^17^	Cross-sectional	8424 schoolchildren	10-17 years	Do not assess	Do not report	Food and nutrition insecurity defined by two structured questions
Martin et al., 2007^15^	Cross-sectional	212 children and 200 parents or tutors	Children from 2 to 12 years	Weight and height	Curves of Centers for Disease Control and Prevention	USDA Food Security Module
Jiménez-Cruz et al., 2007^16^	Cross-sectional	Group in 2001: 1200 children. Group in 2003: 1452 children	Children from 6 to 11 years	BMI and waist circumference	Curves of Centers for Disease Control and Prevention	Community Childhood Hunger Identification Project, adapted for Mexican children
Gundersen et al., 2008^6^	Cross-sectional	841 children and adolescents	3-17 years	Weight and height, BMI, stress	Curves of Centers for Disease Control and Prevention	USDA Core Food Security Module (CFSM)
Chen et al., 2009^18^	Longitudinal	764,526 children	Children born between 1997 and 1999	Low birth weight reported	Not reported	Food and nutrition insecurity assessed by data on low birth weight, economic status, maternal nutritional status and family income
Kirkpatrick et al., 2010^10^	Longitudinal	5809 children and 3333 adolescents	1st group: 10-15 years and 2nd group: 16-21 years	Do not assess	Do not perform	Food and nutrition insecurity assessed by questions asked to the most experienced person
Marjerrison et al., 2011^20^	Cross-sectional	183 families with children and adolescents	<18 yearsMean: 11.8±3.99 years	BMI and A1c hemoglobin	Do not report	Household Food Security Survey Canadian Community Health Survey Module
Sharkey et al., 2012^14^	Cross-sectional	50 mothers and 50 children	6-11 years	Weight, height and BMI	Curves of Centers for Disease Control and Prevention	Tool developed by Connell et al.,^24^ (2004)^a^

a The instrument developed by Connell et al. (2004)^24^ has nine questions directed to the child.

The prevalence of FNiS found in households with children and adolescents ranged from 3.3% to 82%^10^^,^ 14 (Table 2). Several methodologies were employed to identify the FNiS situation. The studies used specific tools to be utilized with families,^6^^,^ 7 ^,^ 13 ^,^ 15 ^,^ 16 tools developed for the answers obtained from children and/or adolescents^14^ and specific and structured questions about food-related issues.^10^^,^ 13 ^,^ 17 In the article by Chen et al.,^18^ food insecurity was assessed through some variables such as birth weight, economic power and time of the year. It was understood that families living in poverty would have greater chance of having FNiS.

**Table 2:** Results found in articles on food and nutrition insecurity and cardiometabolic risk factors in childhood and adolescence.

Author/year	Result association	Ethnicities	Prevalence of FNiS	Limitations
Weinreb et al., 2002^13^	Preschoolers: food and nutrition insecurity and worse health status (*OR*: 2.8), life events (*OR*: 8.5), family size (*OR*: 3.2), low birth weight (*OR*: 1.42). Schoolchildren: food and nutrition insecurity and low birth weight (*OR*: 1.35), health status (*OR*: 3.4), life events (*OR*: 8.8)	Yes	Preschoolers: 59.2% of food and nutrition insecurity	Yes
			Schoolchildren: 66%	
Cook et al., 2004^7^	Food and nutrition insecurity and health status reported as "Fair/poor" (*OR*=1.90; CI: 1.66-2.18). Food and nutrition insecurity and hospitalizations since birth (*OR*=1.31; CI: 1.16-1.48). There was no association between food and nutrition insecurity and growth risk variables (*OR*=1.09; CI: 0.94-1.25)	Yes	21.4% of households with food and nutrition insecurity	Yes
Molcho et al., 2006^17^	Food and nutrition insecurity and lower consumption of fruits (*OR*: 0.66; 95%CI: 0.45-0.87), vegetables (*OR*: 0.68; CI: 0.49-0.87), whole-grain bread (*OR*: 0.66; CI: 0.42-0.90), higher consumption of potato chips among girls and boys (*OR*: 1.62; CI: 1.39-1.85 and *OR*: 1.33; CI: 1.05-1.61 respectively). Food and nutrition insecurity and mental, somatic symptoms (*OR*: 2.42; CI: 2.06-2.78) and emotional symptoms (CI: 1.47; CI: 1.47-1.23)	No	Low social classes: 15.3%	Yes
			Middle class: 15.9%	
			High social classes: 14.8%	
Martin et al., 2007^15^	There was no association between excess weight and food and nutrition insecurity (*OR*: 1.41; CI: 0.67-2.99). Insufficient income and obesity (*OR*: 0.4; CI: 0.18-0.92). Risk of overweight and food and nutrition insecurity (*OR*: 1.34; CI: 0.53-3.36)	Yes	51.4% of households in food and nutrition insecurity	Yes
Jiménez-Cruz et al., 2007^16^	Higher food and nutrition insecurity in children of parents of Native ethnicity (68%; *p*<0.001)	Yes	46% in 2001 group	No
	Higher food and nutrition insecurity in children younger than 9 years (71%; *p* <0.001)		58% in the 2003 group	
	Children without abdominal obesity and higher prevalence of food and nutrition insecurity (78%, *p* <0.001)			
Gundersen et al., 2008^6^	Stress and food and nutrition insecurity at family level (*OR*: 0.05; CI: -0.27 to 0.37), food and nutrition insecurity and cumulative stress (*OR*: 0.02; CI: -0.01 to 0.005)	Yes	44.5% of households with food and nutrition insecurity	Yes
Chen et al., 2009^18^	Food and nutrition insecurity and diabetes mellitus (*OR*: 1.87), inherited metabolic disorders (*OR*: 1.94), iron-deficiency anemia (*OR*: 2.68) and poorly defined symptoms related to nutrition, metabolism and development (*OR*: 2.02)	No	Food and nutrition insecurity value is not shown, the study associates income to food and nutrition insecurity	Yes
Kirkpatrick et al., 2010^10^	Food and nutrition insecurity and higher chances of having worse health status (*OR*=1.91; CI: 1.33-2.74)	No	10-15 years: 3.3%	Yes
	Food and nutrition insecurity was not associated to diagnosed chronic health conditions ( *OR* =1.22; CI: 0.75-1.99)		16-21 years: 3.9%	
Marjerrison et al., 2011^20^	Food and nutrition insecurity and higher rate of hospitalization (*OR*, 3.66; CI: 1.54-8.66). Mean concentration of A1c hemoglobin was higher in children with food and nutrition insecurity	No	21.9% of food and nutrition insecurity	Yes
Sharkey et al., 2012^14^	Food and nutrition insecurity higher total consumption of energy, calcium, calories from added sugars (*β*=4.8. Standard error=2.2. *p*=0.032; *β*=4.4. Standard error=1.9. *p*=0.028 and *β*=8.4. Standard error=2.0. *p*<0.001)	No	82% of children with food and nutrition insecurity	Yes
	Body mass index was not associated with food and nutrition insecurity status a			

a Data not shown.

The publications were heterogeneous regarding the assessed age groups, which ranged from observations of FNiS from birth to 17 years, as well as the established sample sizes, which varied from 50 to 764,526 individuals.^14^^-^ 16 Of the studies that assessed the age ranges related to childhood and adolescence, none considered the observations separately by groups of children and adolescents, as they differ regarding growth, development and maturation status.^19^ These differences can influence the presence of cardiometabolic risk factors.

Only six studies mentioned the use of anthropometric variables that would contribute to identify the nutritional status associated with food insecurity.^6^^,^ 7 ^,^ 14 ^-^ 16 ^,^ 20 Among the assessed anthropometric variables, only height, weight, body mass index (BMI) and waist circumference were mentioned. Martin et al.^15^ used the children's weight associated with parental weight to identify cardiometabolic risk factors (excess weight), in order to assess whether children that had obese parents would be more likely to have excess weight and whether this association would be defined by family characteristics or caused by exposure to FNiS. Sharkey et al.^14^ observed that the BMI of children and adolescents was not associated with FNiS. Regarding the waist circumference (WC), Jiménez-Cruz et al.^16^ found that children without abdominal obesity had higher FNiS prevalence (78%) when compared to those with appropriate WC (22%).

Half of the study classified the assessed population into ethnic groups.^6^^,^ 7 ^,^ 13 ^,^ 15 ^,^ 16 The identified ethnicities were Hispanics, Caucasians, indigenous and non-indigenous populations, black and white ethnicities. In the study by Weinreb et al.,^13^ the population classified as Hispanic had a higher prevalence of severe FNiS. Cook et al. (2004)^7^ found that Hispanics had higher FNiS values (31.2%) and Jiménez-Cruz et al.^6^ observed that children identified as of indigenous ethnicity were more likely to live with FNiS.

Of the assessed articles, nine reported limitations to identify the association mechanism between FNiS and the presence of cardiometabolic risk factors.^6^^,^ 7 ^,^ 10 ^,^ 13 ^-^ 15 ^,^ 17 ^,^ 18 ^,^ 20 The analyses made by the publications showed that the association between food insecurity and at least one cardiometabolic risk factor was identified in nine articles. However, Martin et al.^15^ found no association between FNiS and the analyzed variables (Table 2).

Molcho et al.^17^ found an association between FNiS and consumption of an unhealthy diet, in which the population with food insecurity had lower consumption of fruits, vegetables and fiber, and higher intake of fat. Cook et al.^7^ observed that children living with FNiS were more likely to have an impaired health status and hospitalizations due to the presence of acute/chronic diseases. Chen et al.^18^ demonstrated that children and adolescents living in poverty required more outpatient care due to diseases related to metabolism, nutritional deficiencies and diabetes mellitus.

The association between FNiS and stress or anxiety levels experienced by the families was also present and showed to be related to the constant concern about adequate access to food.^6^

All studies differed in relation to their objectives. They had in common only the subject of FNiS and the assessment of at least one cardiometabolic risk factor. The evaluated associations were FNiS and excess weight,^16^ FNiS and diabetes mellitus,^20^ FNiS and inadequate food consumption,^14^ FNiS and stress.^6^

**Figure 2:**
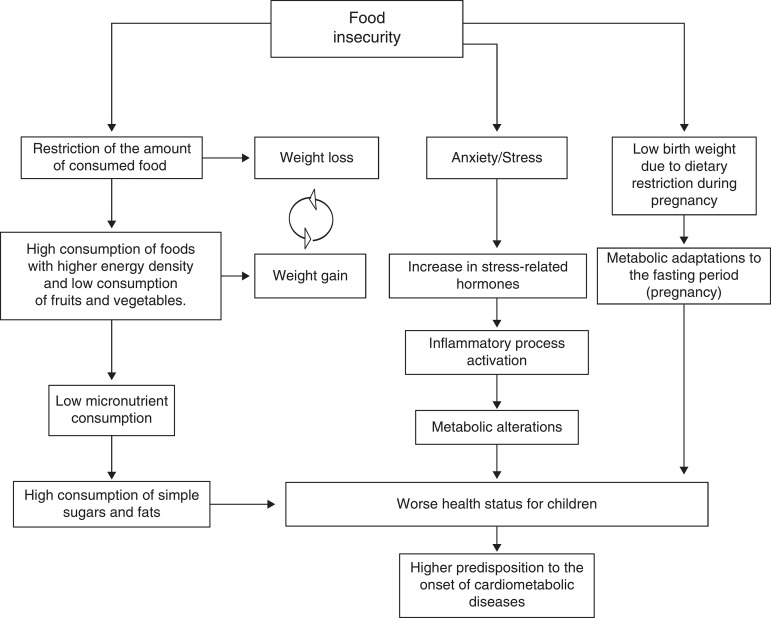
Association between food insecurity and cardiometabolic risk factors in childhood and adolescence.Adapted from Ref. [3].

In an attempt to understand the several mechanisms of the association between FNiS and cardiometabolic risk factors in childhood and adolescence, some possible explanations for this association can be observed (Fig. 2).

## Discussion

The findings of this study show that FNiS may be associated with the presence of cardiometabolic risk factors in childhood and adolescence, such as obesity, stress, metabolic disorders and inadequate dietary patterns.^10^^,^ 16 ^-^ 18

The prevalence of food insecurity among the studies was high.^13^^-^ 16 This prevalence is noteworthy, because for a period of time, the assessed group experienced food deprivation during a phase of life.

Few publications addressed the association between FNiS and cardiometabolic risk factors, especially in children and adolescents. This limitation may be due to the unusual occurrence of chronic diseases in this population. However, some risk factors can be observed and their persistence can lead to the development of some comorbidities. Early diagnosis and treatment are crucial.^21^

There are many possible causes of adverse effects of FNiS in childhood and adolescence. Jiménez-Cruz et al.^16^ report that the presence of FNiS associated with weight alterations early in life might predispose to future risks for obesity, insulin resistance, diabetes, hypertension, high cholesterol levels and metabolic syndrome.

Having a balanced and adequate diet during childhood and adolescence is crucial to decrease health problems.^17^ It is noteworthy that the presence of food insecurity, by itself, can result in risk factors for a worse health status and the development of behavioral problems, such as emotional and psychological stress and anxiety.^7^^,^ 18

Weinreb et al.^13^ point out that the presence of FNiS can result in anxiety and stress for the families. The stress affecting the children can also result in higher levels of diseases. This association is established because the concentrations of stress-related hormones (cortisol, epinephrine, noradrenaline and glucagon) increase during adverse conditions and acute or chronic hypersecretion of these substances can lead to metabolic disorders and inflammation.^22^ Stress also contributes to poor eating habits and lower levels of physical activity, both associated with overweight and obesity, which are risk factors for the development of cardiometabolic diseases.^13^

The study of the association of FNiS with the development of chronic diseases is still scarce, as shown by the articles. Almost all publications in this review draw attention to the difficulty of identifying the mechanisms through which FNiS would increase the risk of developing chronic diseases.^6^^,^ 7 ^,^ 10 ^,^ 13 ^-^ 15 ^,^ 17 ^,^ 20

In an attempt to understand this association, Seligman and Schillinger^3^ reported that FNiS consists in a cyclical factor that turns out to have implications in the incidence of cardiometabolic diseases. In general, families suffering food and nutrition insecurity resort to compensatory strategies during periods of food absence or reduction, which leads to weight loss and hypoglycemia. In times of abundance, there may be excessive consumption of foods that leads to weight gain and hyperglycemia. These behaviors, associated with the state of stress and anxiety, can trigger obesity, hypertension and diabetes.

Martin et al.^15^ emphasize that moments with dietary patterns alternating between the absence or reduction of food with periods of abundance result in metabolic consequences. This situation is related to lower consumption of nutrients, since the consumption of fruits and vegetables decreases and can affect the expression of some chronic diseases triggered by nutrient deficiency.^18^

The heterogeneity of the studies related to age, ethnic groups and FNiS research methodology associated with cardiometabolic risk factors leads to difficulties for comparison and extrapolation of the results to other populations. Most studies carried out in the FNS area are cross-sectional, which does not explain the cause and effect association between the presence of food insecurity and health of children and adolescents.^10^

It is noteworthy the fact that five articles considered the ethnicity of the assessed population, but none explained the significance of this information. Among the many risk factors for the development of cardiometabolic diseases, a positive family history, obesity, physical inactivity, ethnicity and psychosocial factors may have possible associations with and increase the problem.^23^

Considering the complexity and limitations on the understanding of FNiS association with cardiometabolic risk factors, more studies are extremely important for possible reformulations of public health and social policies aimed to reduce the adverse effects of FNiS on health.^18^

It is necessary to increase the knowledge in the food and nutrition insecurity area and recognize the several risk factors this situation brings to the health status of thousands of children and adolescents who live with poverty and hunger. This topic must be present in the offices of health care specialists, who generally do not question and do not assess the food status of their patients and do not consider the association between FNiS and adverse health effects.^13^

## Conclusion

Food and nutrition insecurity is associated with the presence of cardiometabolic risk factors in children and adolescents. Because this is a complex association, some limitations are found to explain the exact mechanism of how the alteration occurs and the direction of such association, as the synergy between the cardiometabolic risk factors the evaluation of heterogeneous groups, the extrapolation of data to other populations and the influence of environmental factors.

The studies showed that food insecurity is associated with worse dietary quality, with reduced intake of fruits and vegetables and increased consumption of refined carbohydrates and fats, micronutrient deficiency, with poor health status and stress situations.

Considering this approach to food and nutrition insecurity, health professionals should be alert to assess the association between FNiS and cardiometabolic risk factors, as well as their consequences for the health of children and adolescents. The identification and early treatment of FNiS and associated risk factors can allow the prevention of future diseases.
